# Spina Bepida Successfully Treated by Iodine Injections

**Published:** 1860-08

**Authors:** Daniel Brainard

**Affiliations:** Prof. of Surgery in Rush Medical College, ECT.


### ARTICLE 2.

### CASE OF SPINA BEPIDA SUCCESSFULLY TREATED BY IODINE INJECTIONS.

BY DANIEL BRAINARD, M. D., PROF. OF SURGERY IN RUSH MEDICAL COLLEGE, ECT.

June 6, 1860, a female child from Michigan was presented to me for treatment. It was eight months old, well formed and healthy in every respect excepting a tumor situated over the upper part of the sacrum. This tumor measured six inches in circumference around the base, eight inches around its largest part, and was elevated two inches above the surrounding skin. Its surface was irregular, resembling that of a tomato, a piece of the colon when inflated. It was translucent elastic at points, and at others the walls were firm like the tissue of cicatrix. A great portion of its contents could be pressed into the spinal column without giving rise to any other inconvenience than making the child cry.

June 6.—Present Dr. Haydock. I passed an exploring trochea into the sac, through a part of the covering, which was thick, and drew off about one ounce of fluid. I then had the neck of the sac pressed on each side and injected through the canula a solution containing $\frac{5}{8}$ gr. of iodine, and $1\frac{1}{2}$ grs. iodide potass, in a drachm dist. water, intending to let it flow back through the canula. This, however, it would not do, and I injected two drams of dist. water, which was allowed to remain. The child, during the operation, was kept under the influence of chloroform. The operation was done at 6 o'clock P. M.

8 o'clock.—Skin hot; child starts in sleep as if frightened; takes the breast.

June 7, 8 o'clock A. M. Has not slept well; has perspired freely; taken the breast; tumor flaccid.

6 P. M.—Seems perfectly well; tumor tense and redder than before the operation.

June 8, 12 M.—Child seems perfectly well; urinates more than natural; tumor tense and red; child laid on the face and side.

June 9.—Tumor red and firm; child perfectly well.

June 10.—Tumor flaccid and pale; applied bands of gum elastic around it.

June 14.—Tumor much reduced. Introduced into a point where the skin is thick and sound, a common hydrocele trochar, and drew off about two drachms of serum tinged with blood from wounding the internal membrane; washed out the cavity with dist. water, and injected the solution used before, as much as could be pressed in, then washed it out with distilled water and applied isinglass plaster.

During the operation a tape was tied tightly around the base of the tumor so as to cut off the connection with the spinal canal, and the child kept under the influence of chloroform.

The operation seemed at first to produce no sensible effect. About an hour afterwards the child had coldness of the feet and hands. This was followed by some reaction and this by sweating. The next day there was some redness and fullness of the tumor which had entirely lost its elasticity.

The patient was seen by Drs. Powell and Paoli.

For three days the tumor was tense, firm and red. After that time it became pale and flaccid.

June 25.—Applied pressure by a gum elastic band around the body, and a band of the same material around the tumor. While this remained it was reduced to about one-third its former size.

June 30.—The tumor is pale, wrinkled, firm, not fluctuating; appears quite solid. Pressure continued by a strip of adhesive plaster passed circularly around it, and the gum elastic band made for a hernia truss passed around the pelvis, so as to exercise compression upon it.

July 6.—Tumor forms only an innodular mass diminishing in size. The patient was allowed to return home with directions to continue the compression as long as might be necessary to efface the walls of the sac.

*Remarks.*—In the No. of this journal for September, 1859, I gave a full account of the origin and history of this treatment as applied to spina bepida, up to that date.

The cases then treated numbered but ten. Since that time Dr. Brackett, of Rochester, has had a case under treatment, the result of which has not yet been fully ascertained, but which Dr. Brackett writes me promises to be favorable, as, although very delicate, the child had, thus far, (at the time of writing,) been better from the effect of each injection.

The case above reported is the sixth which I have treated by injections, and is the most favorable, being the first which was uncomplicated by hydrocephalus, or paralysis. It was also more favorable than previous cases, in these respects the tumor was pediculated so that the injection could be readily prevented from entering the spinal canal, and the walls of the tumor were so thick, at points, that a common hydrocele trochar could be used without danger of subsequent leakage or ulceration.

In any similar case, I should desire: 1st, to tap the sac and draw off the serum; 2d, to make compression so as to prevent the iodine from entering the spinal canal; 3d, inject a solution of iodine, of the strength of five grains, and thrice that quantity of iodide of potash to the fluid ounce of distilled water; 4th, withdraw the injection, wash out the sac with distilled water; 5th, re-inject the serum, or fill the sac with distilled water. The puncture should be carefully closed after withdrawing the camula.

When the tumor is not pediculated, so that the solution may be prevented entering the spine, then the rules I gave in the article refered to, seem to me judicious.

In the case herein reported, no symptoms were produced except those of an over-dose of the solution, diaphoresis, and diuresis, and except for the difficulty of getting rid of the firm walls of the tumor after the sac was obliterated, the case was not more difficult to treat than a hydrocele.

